# Nonequilibrium quantum dynamics in SrTiO_3_ under impulsive THz radiation with machine learning

**DOI:** 10.1126/sciadv.adw1634

**Published:** 2025-09-12

**Authors:** Francesco Libbi, Anders Johansson, Boris Kozinsky, Lorenzo Monacelli

**Affiliations:** ^1^ John A. Paulson School of Engineering and Applied Sciences, Harvard University, Cambridge, MA 02138, USA.; ^2^ Robert Bosch LLC Research and Technology Center, Watertown, MA 02472, USA.; ^3^ Department of Physics, Sapienza University of Rome, Piazzale Aldo Moro 5, 00185 Rome, Italy.

## Abstract

Ultrafast spectroscopy paved the way for probing transient states of matter produced through photoexcitation. The microscopic processes governing the formation of these states remain largely unknown, due to the inherent challenges in accessing the microscopic behavior of materials, which is strongly influenced by nuclear quantum effects. Here, we perform simulations of quantum nuclear dynamics in the nonequilibrium regime, extending beyond the current state of the art. By combining first-principles simulations with machine learning, we unveil the complex quantum dynamics of SrTiO_3_ emerging after terahertz laser pumping. We disclose the microscopic origin of the phonon upconversion, observed experimentally but not fully understood, and quantify the lifetime of the out-of-equilibrium motion, which is beyond the reach of the state-of-the-art simplified models. Crucially, our simulations predict that terahertz pump pulses can generate persistent out-of-equilibrium stress capable of inducing polar order. This work lays the foundation for systematic explorations of complex quantum materials sensitive to photoexcitation.

## Teaser

A fast machine-learned approach is used to simulate quantum dynamics in SrTiO₃ driven far from equilibrium by intense THz pulses.

## INTRODUCTION

Advances in ultrafast laser technology enable the generation of pulses that last for as little as one electric field oscillation, providing direct probe of the out-of-equilibrium quantum dynamics of ions. By using ultrashort laser pulses to excite (pump) and subsequently probe the material’s response, it is possible to capture transient dynamics, shedding light on new states of matter (*1*) and allowing the manipulation of macroscopic properties such as polarization, magnetism, and conductivity. Dynamical switching between different states of matter like metal-insulator (*2*, *3*), para-ferroelectric (*4*, *5*), para-ferromagnetic (*6*), topological (*7*), and even superconducting (*8*) have been investigated, pointing to new possibilities for material design and dynamic control and promising transformational advances in technology and applications. On the other hand, intense pulses drive the system out of equilibrium (*9*), illuminating the intrinsic interactions among collective atomic motions by probing the resulting nonlinear dynamics (*10*–*15*). Despite the rapid progress of the field, many of the mechanisms of the aforementioned phenomena are still actively debated. Experimental probes only show a trace of the underlying complex quantum dynamics of ions and electrons, and a deeper theoretical understanding is needed, via computations of realistic materials’ response to experimentally relevant stimuli.

An outstanding example is the dynamic response to laser pulses of the SrTiO_3_ (STO) perovskite. In this material, nuclear quantum fluctuations are observed to suppress the ferroelectric order below 37 K (*16*). This effect is due to the sufficiently small energy barrier of the double-well potential energy surface associated with the ferroelectric soft phonon mode (SPM), which allows the nuclear wave function to span the two minima, leading to tunneling and a consequent nonferroelectric phase, stabilized by quantum fluctuations down to 0 K (*17*, *18*). Recent experiments reported the occurrence of second-harmonic generation (SHG) lasting for at least several picoseconds after an ultrafast excitation with terahertz light (*4*, *5*), which is a signature of the inversion symmetry breaking. This was interpreted as the formation of a ferroelectric metastable phase following the pump pulse (*19*). However, this interpretation has been challenged by a similar observation on KTaO_3_, another quantum paraelectric (*20*), where the appearance of the SHG after the pump was associated with the terahertz-driven dipole correlations of defect-induced local polar structures (*21*). As a result, it remains unclear whether the laser pulse can stabilize a hidden ferroelectric phase and induce a macroscopic polarization.

The theoretical first-principles modeling of the light-induced response is necessary to examine the possible transient ferroelectric transition, but it is highly challenging. The current state-of-the-art approach is to build a low-dimensional model of the system retaining only a few degrees of freedom (the photoexcited vibrational mode and those modes deemed responsible for the occurrence of the light-induced transition), derive their anharmonic coupling from perturbation theory, and numerically determine the quantum dynamics of the model (*19*). While this approach provides fundamental physical insight into the role of coupling between the acoustic modes and the SPM in light-induced ferroelectricity, it requires understanding which phonon modes most substantially influence the dynamics and drive the ferroelectric transition. Identifying these modes is typically feasible postexperiment but remains highly challenging to determine a priori. Furthermore, many existing models in the literature do not directly account for nuclear quantum effects (*10*, *21*, *22*), which crucially affect the structural and dynamical properties of STO. In addition, real-time x-ray spectroscopy has revealed that photoexcitation of the SPM can induce complex energy transfer to high-energy transverse optical (TO) modes on a femtosecond timescale (*10*). This phonon upconversion highlights the need for a fully atomistic description of the dynamics in STO, as multiple degrees of freedom are involved.

An unsupervised, fully atomistic approach is thus required. One method to study atomistic quantum nuclear dynamics from first principles involves real-time path integral formulations (*23*–*26*), but they suffer from the so-called sign problem (*27*). As a result, these methods are applicable to cases where only when a few degrees of freedom interact (*28*) and cannot address realistic quantum dynamics of complex systems. This is due to the difficulty in converging calculations with respect to the number of paths of opposite signs, which exponentially increases with the number of degrees of freedom. An additional problem with such approaches is the difficulty in capturing quantum tunneling phenomena, which is especially important for the dynamics of STO. Imaginary path integral methods avoid some of these problems but are only rigorously formulated for equilibrium statistical properties and cannot address transient light-induced response phenomena (*29*–*33*). Furthermore, their computational cost diverges as the temperature approaches absolute zero. Therefore, there is a need for an efficient nonperturbative simulation method applicable at low temperatures and capable of capturing real-time out-of-equilibrium quantum dynamics of the nuclei.

## RESULTS AND DISCUSSION

In this work, we use an advanced methodology for efficiently solving the quantum nuclear Schrödinger equation and compute the nonlinear dynamics of STO under intense pulsed terahertz radiation from first principles without any assumption on the phonon interactions. This is achieved by formulating a rigorous time-integration scheme (*34*) for calculating the quantum nuclear dynamics through the time-dependent self-consistent harmonic approximation (TDSCHA) (*35*, *36*) combined with an efficient description of the Born-Oppenheimer surface using a state-of-the-art machine learning force field (*37*, *38*) trained on density functional theory (DFT).

**Fig. 1.**
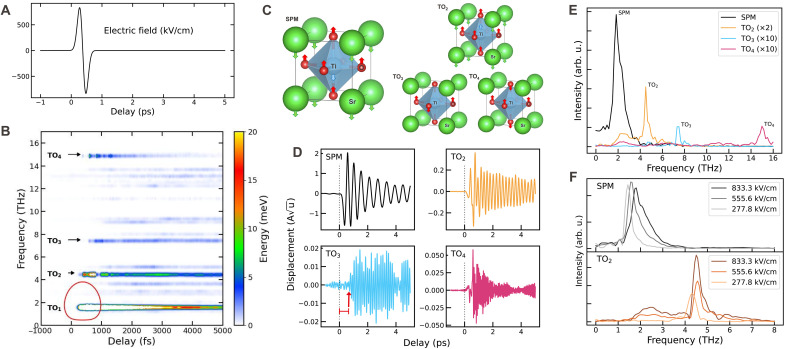
Phonon upconversion. (**A**) Pulse applied to STO. (**B**) Phonon energy as a function of time and its natural frequency. (**C**) Schematic representation of the four groups of optical modes at Γ . (**D**) Γ-phonon displacement as a function of the pulse delay. (**E**) Phonon spectral density, obtained as the Fourier transform of the displacements reported in (D). (**F**) Dependence of the phonon spectral density as a function of the pulse amplitude; the signals have been zero-padded and windowed for processing.

We start the simulation at time t=−1000 fs using a 40-atom supercell of STO, thermally equilibrated at 100 K to match the experimental temperature reported by Kozina *et al.* (*10*). This initial state is achieved via the stochastic self-consistent harmonic approximation (SSCHA) (*39*), an equilibrium approach already validated for correctly capturing the quantum suppression of ferroelectricity in STO (*18*). Then, at t=0 fs, the system interacts with a single-cycle electric field in resonance with the SPM (the terahertz laser pump, shown in Fig. 1A), and is driven out of equilibrium. The ability to generate such a single-cycle oscillation of the electric field has only recently become possible due to advances in control of terahertz fields (*10*). The dynamical coupling between the laser light and the nuclear coordinates is determined by the Born effective charges, evaluated within DFT at the equilibrium structure and assumed constant throughout the simulation. The quantum nuclear density matrix is evolved according to forces obtained from the machine learning interatomic potential (*37*, *38*) (more details in Materials and Methods) and additional force contributions due to light-matter interaction for the first few hundreds of femtoseconds (pulse duration). Following the experimental setup of Kozina *et al.* (*10*), the electric field was oriented along the [1,−1,0] crystallographic direction of STO’s reference cubic cell. We set the field’s maximum amplitude to 833 kV/cm.

At 100 K, the primitive cell is nearly cubic (the antiferrodistortive transition occurs at 105 K), wherein the phonons at the Γ point are organized into four distinct groups of degenerate modes, represented in Fig. 1C. The first group (TO_1_) includes the SPMs with a frequency of 1.5 THz, which are responsible for the ferroelectric transition when nuclear quantum fluctuations are suppressed [e.g., through ^18^O substitution (*40*)]. The remaining groups of degenerate phonons have frequencies 4.8, 7.7, and 15.4 THz. These frequencies have been computed as eigenvalues of the free energy Hessian, following (*41*), and will be referred to as natural frequencies of the phonons. The phonon modes polarized along the direction of the electric field are labeled respectively as TO_2_, TO_3_, and TO_4_.

Shortly after the pump interacts with the system and the SPM is excited, the phonon-phonon interactions transfer the energy in a complex way between all 117 phonon modes compatible with our simulation cell. To examine how energy flows from the excited SPM to the other degrees of freedom, we decomposed the total energy into the time-dependent contributions of individual phonons, reported in Fig. 1B (implementation details are in Materials and Methods). Approximately 300 fs after the pump arrives, the TO_2_, TO_3_, and TO_4_ modes begin oscillating, as shown in Fig. 1D. This process has been observed for the TO_2_ and TO_3_ modes by time-resolved x-ray diffraction (*10*) and is referred to as phonon upconversion.

After 1.0 ps, the anharmonic phonon scattering transfers energy to modes at finite momenta. We observe a background of almost all modes that gradually acquire energy, damping the motion generated by the pulse. This is an effect of dissipation that results into thermalization of the system.

**Fig. 2.**
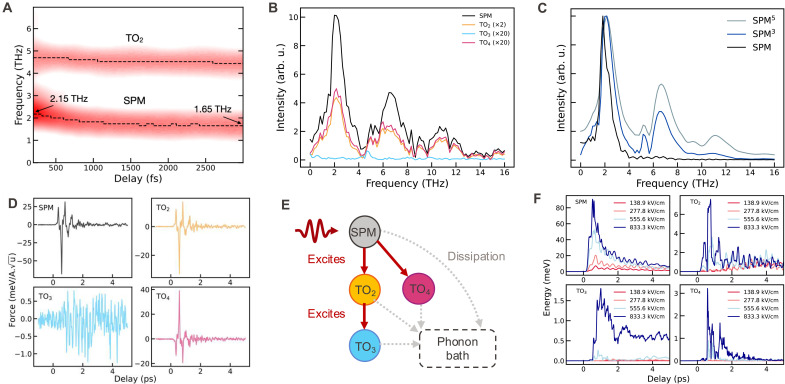
Upconversion interpretation. (**A**) Time dependence of the SPM and TO_2_ phonon modes. (**B**) Fourier transform of the anharmonic force projected on the phonon modes at Γ . (**C**) Fourier transform of the coordinate of the SPM mode, raised to different powers. (**D**) Time-dependent anharmonic force projected on the phonon modes at Γ . (**E**) Schematic representation of the energy flow between phonon modes. (**F**) Energy of the Γ-phonons as a function of the amplitude of the electric field.

To gain a better insight into the process of the upconversion, we calculate the Fourier transform of the Γ-modes oscillations. As depicted in Fig. 1E, the vibrational spectrum of modes TO_2_, TO_3_, and TO_4_ peaks at their natural frequency. However, the SPM oscillates at a higher frequency (~2.0 THz instead of its natural frequency of 1.5 THz) due to the stiffening caused by the anharmonic terms in the potential energy surface of the SPM when driven to large oscillations by large electric fields. Such stiffening as a function of the amplitude of the electric field is illustrated in Fig. 1F. The broadening of the spectrum with increasing pulse intensity is associated with a reduction in the SPM lifetime due to the increased energy transfer from the SPM to the phonon bath, which will be analyzed in more details later in the main text. The stiffening of the phonon frequencies of the SPM and TO_2_ modes is time dependent, as shown in Fig. 2A, which was obtained by computing the Fourier transform of the displacements in Fig. 1D using a moving window of width 1.5 ps. This behavior arises from the fact that, initially, both modes have sufficient energy to explore the anharmonic regions of their potential energy surfaces. As energy dissipates over time, the dynamics become confined to the near-harmonic regime. The substantial time-dependent renormalization of the SPM frequency (~40%) is particularly noteworthy and encourages further investigation using time-resolved techniques such as pump-probe Raman spectroscopy.

To unveil the mechanism of the excitation of the TO_2_, TO_3_, and TO_4_ modes, we plot in Fig. 2B the Fourier transform of the average anharmonic force projected on the atomic motion of each mode μ . This is obtained by subtracting the harmonic restoring force $-\omega_{\mu }^{2}Q_{\mu }$ from the total average force acting on the phonon mode. Here, $\omega_{\mu }$ refers to the phonon natural frequency, while $Q_{\mu }$ refers to its displacement amplitude (details in Materials and Methods). The anharmonic force spectrum for the modes SPM, TO_2_, and TO_4_ is characterized by three prominent peaks with amplitudes decreasing linearly as a function of frequency. The ratio of the peak heights matches very well with that of the fifth power of $Q_{\text{SPM}}$ , which is illustrated in Fig. 2C. This suggests that high electric fields (833 kV/cm) excited the SPM sufficiently strongly to drive the system far from equilibrium into a highly anharmonic part of the free energy surface. A much different case is that of the TO_3_ mode, whose anharmonic force spectrum is peaked at the TO_2_ frequency, suggesting that it is excited through the TO_2_ mode. The plot of such forces in time (Fig. 2D) shows that the sudden oscillation of the SPM far from equilibrium generates an impulsive force on the TO_2_ and TO_4_ modes through anharmonic coupling. The coherent oscillations of TO_2_ then drive the TO_3_ mode (see Fig. 2E for a schematic representation).

To further delve into the process of the phonon upconversion, we analyze the response of the Γ phonon modes while varying the amplitude of the pump pulse. Figure 2F illustrates the evolution of the energy of these modes as a function of the pulse amplitude when it is in resonance with the frequency of the SPM mode at 100 K. The responses of these modes increase nonlinearly with the amplitude of the electric field. The energy of oscillation of the higher-frequency TO modes correlates with that of the SPM. Notably, we observe almost complete suppression of phonon upconversion for amplitudes below 277.8 kV/cm, further indicating the nonlinear character of this process. For an electric field intensity of 833.3 kV/cm, the maximum energies of the TO_2_, TO_3_, and TO_4_ modes are ~7, 1.5, and 3 meV, respectively, indicating that a considerable fraction of energy is upconverted into the TO_3_ and TO_4_ modes despite their relatively small displacement amplitudes. This behavior arises from the fact that the energy of a phonon mode scales with the square of its frequency. Consequently, a minimal model describing the phonon upconversion must necessarily include the TO_3_ and TO_4_ modes, despite their small oscillation amplitudes.

The full atomistic descriptions of the system enable us to access the lifetime of the phonon mode excitation, as the large number of degrees of freedom acts as the thermal reservoir into which the energy of the excited subsystem eventually decays. In particular, our simulations did not use any empirical smearing or dephasing factor, thus providing the real lifetime arising from the natural anharmonic phonon scattering among the atomic degrees of freedom. The lifetime of the SPM is strongly affected by the pump intensity. Previous experimental results showed that for applied pulses with amplitude below 80 kV cm^−1^, the SPM lifetime increases with the laser intensity (*42*), manifested as a spectral narrowing. However, the onset of phonon upconversion at amplitudes around 280 kV cm^−1^ points to the appearance of new pathways for energy to decay. The SPM lifetime further decreases upon increasing pump amplitude, thus unveiling a complex dependence of the transient dynamic lifetimes with the pulse intensity. The ability of our explicit, fully atomistic dynamic simulation to capture amplitude-dependent damping of the SPM mode goes beyond previous models that assumed an effective constant damping factor (*10*, *19*, *21*, *22*, *43*).

**Fig. 3.**
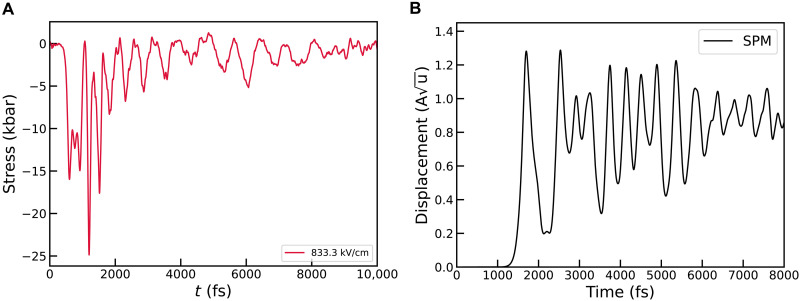
Induced stress and ferroelectric transition. (**A**) The stress induced on STO by the irradiation with a terahertz pulse of amplitude 833.3 kV/cm. (**B**) Symmetry breaking of the SPM mode.

There is now a vigorous debate about the origin of the SHG signal observed in the experiments in (*4*, *5*). In (*5*, *19*), lattice strain has been identified as the factor responsible for such a ferroelectric transition. STO in equilibrium at low temperature is paraelectric but very close to a ferroelectric instability, with the paraelectric structure stabilized by the quantum zero-point motion of nuclei (*18*). A positive strain increases the barrier between the two minima in STO’s potential energy surface, thereby potentially stabilizing the ferroelectric phase. To determine whether a metastable state of the free energy as a function of strain exists—potentially justifying a permanent ferroelectric transition—we perform SSCHA relaxations on a 20-atom STO cell across a range of isotropic strains from −0.3 to 0.9% at 0 K. We observed that a strain of 0.1%, corresponding to a stress of few hundreds kbar, is sufficient to destabilize the paraelectric phase and induce a ferroelectric transition. Details of the calculation are in the Supplementary Materials. As the strain increases further, we observe a monotonic rise in free energy, suggesting that no ferroelectric metastable state with periodicity in the 20-atom cell appears in the free energy–strain diagram. This implies that a static metastable ferroelectric distortion, if any, may occur on a larger periodicity. A dynamical ferroelectric transition remains possible, induced by the nonequilibrium transient strain resulting from the system’s dynamic response to a light pulse. To verify this occurrence, we perform TDSCHA simulations where the system is prepared at 0 K and pumped with a pulse polarized along the *c* axis with a maximum field of 833 kV cm^−1^. Our TDSCHA approach at this point is only applicable at fixed volume and does not allow for the exploration of dynamic strain effects and any consequent ferroelectric transitions that may emerge as a result, unless extremely large cells are adopted. However, we can obtain indirect evidence of such transitions by analyzing the instantaneous stress acting on the cell, accounting for the quantum fluctuations of the out-of-equilibrium dynamics using the formalism introduced in (*44*). When the pump interacts with the sample, a massive stress of 2.5 GPa is induced along the *c* axis, as shown in Fig. 3A. After the end of the pump, the stress persists and oscillates around negative values (corresponding to tensile stress in our convention), at a frequency that is twice that of the SPM at 0 K, which ranges from 0.6 to 1.0 THz (because of the stiffening due to anharmonicity, as discussed above). This residual stress is substantial, with peak values reaching 0.5 GPa. If this stress were to induce a strain of the cell, it would be sufficient to generate a ferroelectric dynamical phase transition, which could persist as long as the atomic motion has enough energy to sustain the strain in the system. We verify this by repeating the simulations for a cell strained along the *z* direction, corresponding to a stress of 0.5 GPa (see Fig. 3B). After irradiation with a terahertz pulse of 250 kV/cm, the SPM begins oscillating around a symmetry-broken state, with the oscillation amplitude gradually decreasing due to energy exchange with other modes, confirming the occurrence of a ferroelectric transition. The propagation of strain would occur as acoustic waves within the material. In a perfectly bulk material, the time required for the strain to manifest is excessively long, incompatible with the picosecond timescale of the transition observed in (*4*). However, the presence of polar nanoregions stemming from defects in STO (*21*), spaced at an average distance of nanometers, could markedly reduce this time, thereby rendering it compatible with a picosecond timescale. Specifically, this time corresponds to the duration needed for an acoustic wave with a group velocity of ~10^3^ m/s to traverse nanometer-scale boundaries. A strain-mediated mechanism for the light-induced ferroelectric transition was proposed by Shin *et al.* (*19*), where, however, the presence of few degrees of freedom makes the ferroelectric state metastable, thus resulting in a signal for SHG that persists indefinitely. This did not allow the comparison between the lifetime of the ferroelectric state with the available experimental data. We argue that inversion symmetry breaking does not correspond to the presence of a metastable or thermodynamically stable phase in the free energy–strain diagram. Instead, ferroelectricity only exists as a dynamical transient out-of-equilibrium state that can persist only as long as the atomic motion maintains enough stress to deform the local potential into a sufficiently deep double well, allowing the spontaneous breaking of inversion symmetry.

**Fig. 4.**
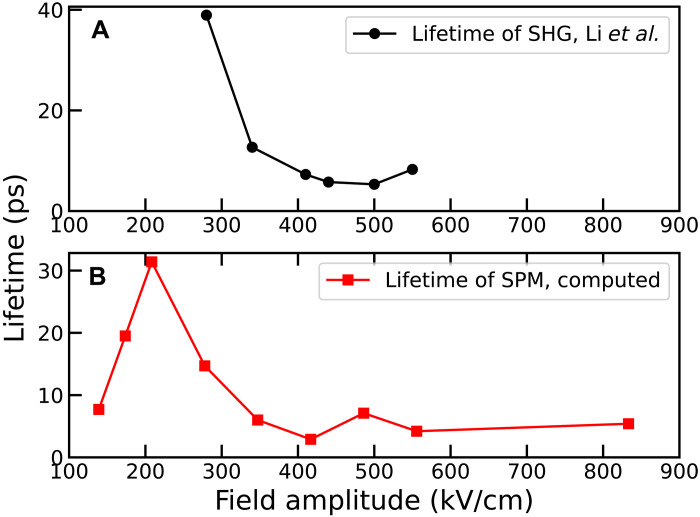
Lifetimes. (**A**) Lifetime of the SHG extracted from the measurements in (*4*). (**B**) lifetime of the SPM calculated at 100 K.

Furthermore, we observe that, because of the increase of the anharmonic phonon scattering above 200 kV/cm, a more intense pump field results in a shorter lifetime of the SPM and, thus, of the dynamical stress. In Fig. 4 (A and B), we compare the simulated lifetime of the SPM as a function of the electric field amplitude at 100 K, which is a good probe of the duration of the transient nonlinear dynamics, with the measured lifetime of the inversion symmetry breaking observed by SHG after terahertz pulse at low temperature (*4*). Both lifetimes show a strong correlation above 270 kV cm^−1^, the field value corresponding to the onset of the phonon upconversion. The lifetimes of both the SHG and the SPM decrease with increasing field amplitude. This is the opposite of the trend reported by Cheng *et al.* (*21*) for the KTaO_3_ perovskite, where the inversion symmetry breaking was associated with terahertz-driven dipole correlations of defect-induced local polar structures. On the other hand, in STO, the strong correlation of the SHG signal with the lifetime of the SPM suggests that the SHG signal is brought about by complex out-of-equilibrium lattice dynamics induced by the electric field pulse.

In conclusion, thanks to an advanced framework for simulating the out-of-equilibrium quantum nuclear dynamics from the first principles, we unveil the origin of the complex nonlinear phenomena observed in STO under intense terahertz laser pulses. We disclose the microscopic mechanism of the phonon upconversion and the complete energy decay pathway across different phonons, both coherent and incoherent, following laser excitation. Thanks to a parameter-free description, we identified a fingerprint of the ferroelectric transition in the light-induced stress calculated in STO, substantial enough to induce a polar order. Our advanced technique for the nuclear quantum dynamics paves the way for a fully first-principles investigation and identification of new materials that can host light-induced phase transitions.

## MATERIALS AND METHODS

The nonlinear quantum dynamics of nuclei is simulated through the TDSCHA (*35*). The TDSCHA assumes that the nuclear quantum density matrix is Gaussian in the Wigner phase-space (*45*, *46*), parameterized by the mass-rescaled position-position, momentum-momentum, and position-momentum covariances A=〈δRδR〉 , B=〈δPδP〉 , and Γ=〈δRδP〉 and centered at the average atomic positions R and momenta P . TDSCHA represents the dynamic generalization of the SSCHA, which has proven to describe very well the vibrational and structural properties of different categories of anharmonic materials and their temperature dependence (*39*, *47*–*49*). In particular, SSCHA reproduces the structural properties of perovskites very accurately (*50*) and predicts the correct quantum paraelectric phase for STO (*18*). Furthermore, the linear response of TDSCHA has allowed for an unprecedented description of the Raman and infrared spectra of metallic hydrogen (*51*). The dynamics for the parameters R , P , A , B , and Γ are obtained by imposing the least action principle (*36*), leading to the self-consistent time-dependent equation for the density matrix

$$i\hbar \frac{\partial \hat{\rho }}{\partial t}=\left[H\left[\hat{\rho }\right],\hat{\rho }\right]$$ (1)

where $\hat{\rho }$ is the nuclear quantum density matrix, $H\left[\hat{\rho }\right]$ is the self-consistent harmonic Hamiltonian whose parameters depend on the anharmonic potential and the density matrix $\hat{\rho }$ at the same time, and the square brackets denote the quantum commutator (*35*). Substituting the Gaussian expression for the density matrix leads to the set of differential equations

$$\left\{\begin{array}{c} \dot{R}=P\\ \dot{P}=\langle f\;\rangle \\ \dot{A}=\Gamma +\Gamma^{†}\\ \begin{array}{c} \dot{B}=-\langle \partial^{2}V\rangle \Gamma -\Gamma^{†}\langle \partial^{2}V\rangle \\ \dot{\Gamma }=B-A\langle \partial^{2}V\rangle . \end{array} \end{array}\right.$$ (2)

We assume that the terahertz pulse does not excite electrons to higher energy states, as the bandgap of STO (3.2 eV) is substantially larger than the terahertz photon energy. Consequently, the ionic motion is described by the ground-state Born-Oppenheimer potential energy surface.

The dynamic simulations are substantially accelerated using a machine-learned interatomic potential trained with state-of-the-art active learning strategies. This active learning is conducted over a run of 1000 ps, covering a range of strains from −2 to 2% and temperatures up to 500 K.

The potential is trained on DFT calculations with the PBE functional, as described in detail in the first section of the Supplementary Materials. According to the work of Verdi *et al.* (*18*), this results in an underestimation of the barrier height of the potential energy surface of the SPM (third section of the Supplementary Materials). Such underestimation leads to an increase of the frequency of the ferroelectric soft mode, which in our calculations is ν=2.6 THz at 100 K and ν=1.5 THz at 0 K, compared to the experimentally determined ν∼1.5 THz at 100 K (*10*) and ν∼0.5 THz at 0 K (*52*). To address this limitation, we apply a uniform strain to the STO cell. The strain value is chosen to match the experimental frequency of the SPM as obtained by diagonalizing the free energy Hessian of STO. The adopted strain values are then 0.3% at 100 K and 0.5% at 0 K. These very small strains are expected to leave the coupling constant between phonon modes unchanged, as we tested by observing negligible frequency changes in all other modes. However, they induce a sizable correction in the frequency of the SPM due to its extreme sensitivity to the shape of the double-well potential energy surface.

The pulse shape used to study the phonon upconversion is modeled as

$$f\left(t\right)=-A\;\frac{t}{\sigma }e^{-\frac{t^{2}}{2\sigma^{2}}+\frac{1}{2}}$$ (3)

with $\sigma =1/\left(2\pi f_{\text{SPM}}\right)≃$ 0.1 ps (more details in the Supplementary Materials).

To analyze the energy transfer between different vibrational modes, we project the atomic displacements onto the phonon modes computed at the equilibrium at 100 K by diagonalizing the free energy Hessian (*41*), using the following relation

$$Q_{\mu }=\sum_{a}{\sqrt{m}}_{a}e_{\mu a}u_{a}$$ (4)

where μ is the phonon branch index, a labels both atoms in the supercell and Cartesian coordinates, $e_{\mathrm{\mu a}}$ is the phonon eigenvector at equilibrium, and $u_{a}=R_{a}\left(t\right)-R_{a}\left(0\right)$ represents the displacement of the centroid of the Gaussian from the equilibrium position. We define $h_{\mu }\left(t\right)$ as the out-of-equilibrium local energy of the μ th phonon

$$h_{\mu }\left(t\right)=\frac{{\dot{Q}}_{\mu }^{2}}{2}+\frac{\omega_{\mu }^{2}Q_{\mu }^{2}}{2}$$ (5)

where the two terms in the right-hand side represent the phonon kinetic energy and harmonic elastic energy, respectively. The sum of $h_{\mu }$ on all modes is the centroid’s energy. To obtain the system’s total energy, we also consider the effects of the quantum-thermal dispersion and the anharmonicity (*36*). These effects arise from the interactions among many phonons and cannot be repartitioned on a mode-by-mode basis. However, these contributions are almost constant during the motion; thus, $h_{\mu }\left(t\right)$ is a good representation of the energetic contribution of each mode. The energy spectral density

$$h\left(\omega ,t\right)=\sum_{\mu }h_{\mu }\left(t\right)\delta \left(\omega -\omega_{\mu }\right)$$ (6)

is shown in Fig. 1B. The area outlined in red indicates the spectrogram of the terahertz pump pulse (contour at 30% of peak magnitude). The choice to project the dynamics onto the initial equilibrium phonon modes, rather than time-dependent ones, is purely a postprocessing decision and does not introduce any approximation in the SSCHA time evolution, which self-consistently evolves all physical quantities, including the phonons. The purpose of Eq. 5 and Fig. 1B is to provide an estimate of the energy transferred to the high-frequency modes. The time dependence of the phonon modes is not considered in this postprocessing analysis, as their calculation in a nonequilibrium regime is inherently nontrivial and requires the evaluation of nonlinear susceptibility functions, which lies beyond the scope of the present work. An approximate calculation of the time-dependent phonon frequencies for the SPM and TO_2_ modes is presented in Fig. 2A. Nonetheless, including the time dependence of the phonon modes in Eq. 5, while offering a more complete picture, is not expected to substantially affect the estimate of the energy transferred to the high-frequency modes.

The average force acting on the mode μ can be calculated as

$$f_{\mu }=\sum_{i}\frac{{\langle \;f\rangle }_{i}}{\sqrt{m_{i}}}e_{\mu i}$$ (7)

The expected anharmonic force is obtained as

$$f_{\mu }^{\text{anh}}=f_{\mu }+\omega_{\mu }^{2}Q_{\mu }$$ (8)

the first term is the total force acting on the mode μ , and the second subtracts the harmonic force. $f_{\mu }^{\text{anh}}$ is the quantity represented in Fig. 2D of the main text.

The effective charges describe the interaction between laser light and nuclear coordinates, accounting for the dielectric screening of local fields, as discussed in the second section of the Supplementary Materials.

**Fig. 5.**
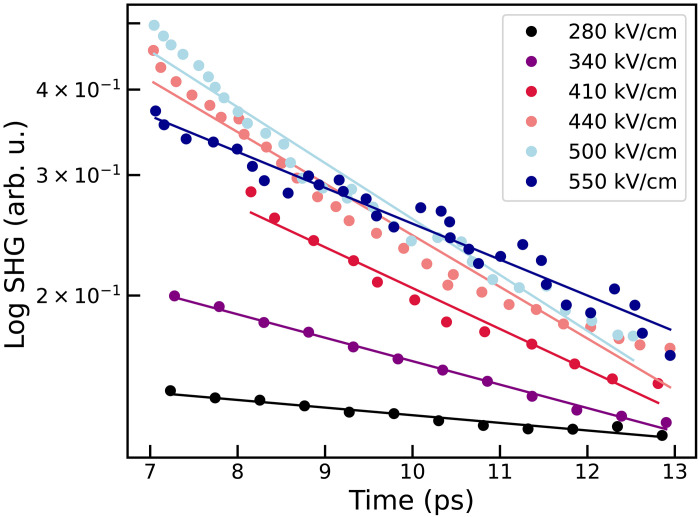
SHG signal Fit of the SHG signal from Fig. 2C of (*4*).

The experimental lifetimes shown in Fig. 4B were obtained by fitting the data from figure 2C of (*4*) in the time window between 7 and 13 ps, where the transient response to the pulse has subsided and the signal exhibits a clear exponential decay. The upper limit of 13 ps was chosen because of difficulties in accurately extracting the experimental data beyond this point. For the same reason, it was not possible to reliably extract the values corresponding to 170 and 230 kV/cm, as the curves are very close to each other and approach zero. The logarithm of the data in this region was fitted with a straight line (see Fig. 5); the inverse of the slope of this fit corresponds to the extracted lifetime.

## Supplementary Materials

### This PDF file includes:

Supplementary Text Figs. S1 to S5 Table S1 References
