# Supplementary material for: Nonequilibrium quantum dynamics in SrTiO3 under impulsive THz radiation with machine learning
Source: Sci Adv. 2025 Sep 12;11(37):eadw1634. doi: 10.1126/sciadv.adw1634 (PMC13141898; doi:10.1126/sciadv.adw1634)
Supplement: Supplementary file 1 — Supplementary Text Figs. S1 to S5 Table S1 References [file sciadv.adw1634_sm.pdf]

Science Advances
AAAS

Supplementary Materials for

Nonequilibrium quantum dynamics in $SrTiO_3$ under impulsive THz radiation with machine learning

Francesco Libbi *et al.*

Corresponding author: Francesco Libbi, libbi@g.harvard.edu; Lorenzo Monacelli, lorenzo.monacelli@uniroma1.it

Sci. Adv. **11**, eadw1634 (2025)
DOI: 10.1126/sciadv.adw1634

This PDF file includes:

Supplementary Text
Figs. S1 to S5
Table S1
References

I. MACHINE-LEARNED POTENTIAL

For the machine-learned interatomic potential (MLIP), we choose FLARE [53], as it provides active learning for efficient data generation and fast inference times [54]. The active learning explored different temperatures and volumes to create a transferable potential. At each of 100, 300 and 500 K, the system was simulated for 200 ps at the DFT-relaxed lattice parameter as well as ±2% strain for a total of 1.8 ns of dynamics. We used a timestep of 2 fs and a thermostat damping time of 200 fs with LAMMPS's [55] default Nosé–Hoover thermostat. The procedure is shown in FIG. S1, where the bottom panel shows how the maximum atomic uncertainty was used to determine whether to compute forces, energy and stress with DFT and retrain the potential. Before each change in temperature, the uncertainty threshold was doubled. The DFT calculations are performed at the PBE level of theory, using the open source software Quantum ESPRESSO [56]. We employ a plane-wave cutoff of 80 Ry, and a k-point grid of $6 \times 6 \times 8$ for the 20-atom cell. We adopt the pseudopotentials suggested by the SSSP efficiency library [57].

The double well potential energy given by the MLIP is in good agreement with the density functional theory results (see Fig. S2). In order to check the accuracy of the MLIP, we calculate also the equation of state, both for the cubic and the tetragonal cells, reported in Fig S3. Being short-ranged, the MLIP is not able to capture long-range effects in the force constants. As a result, it does not fully capture the splitting between the high-frequency longitudinal and transverse optical degenerate modes [58–61]. This error in the phonon frequencies is not expected to significantly affect the dynamics, as the SPM is characterized by a much lower frequency.

II. PULSE SHAPE, DIELECTRIC SCREENING AND BORN EFFECTIVE CHARGES

To simulate phonon upconversion, we use a single-cycle pulse of the following shape, which reproduces the main features of the pulse applied in Ref. [10]:

$$f(t) = -A\,\frac{t}{\sigma} e^{-\frac{t^2}{2\sigma^2}+\frac{1}{2}} \tag{S1}$$

This pulse reaches its maximum at $t = \sigma$, with $|f(\sigma)| = A$. Furthermore, its Fourier transform is

$$F(\omega) = -iA\,\sigma^2\omega\; e^{-\frac{1}{2}\omega^2\sigma^2+\frac{1}{2}} \tag{S2}$$

which peaks at $\omega_0 = 1/\sigma$, corresponding to a frequency of

$$f = \frac{1}{2\pi\sigma} \tag{S3}$$

The investigation of the THz field induced ferroelectric transition, instead, employ a Gaussian pulse of equation

$$f(t) = A\cos(\omega_0(t - t_0))e^{-\frac{(t-t_0)^2}{2\sigma^2}} \ . \tag{S4}$$

The value of σ employed in the simulations is 468 fs. The Fourier transform of this pulse reads

$$F(\omega) = \frac{\sigma}{2}\left(e^{-\frac{1}{2}(\omega-\omega_0)^2\sigma^2} + e^{-\frac{1}{2}(\omega+\omega_0)^2\sigma^2}\right) \ . \tag{S5}$$

If $\omega_0 > \sigma$, this pulse is peaked at $\omega = \omega_0$, with spread $1/\sigma$. The forces on the atom due to the electric field are obtained as

$$\mathbf{f}_a = \frac{1}{\varepsilon_{eff}} \, \mathbf{Z}_a \cdot \mathcal{E} \ , \tag{S6}$$

where $\mathcal{E}$ is the external electric field, ε_{eff} is the dielectric constant and $\mathbf{Z}_a$ are the Born effective charge tensors. The effective charges are calculated through Density funtional perturbation theory (DFPT), using the same parameters as specified in Section I. Their value for the different atomic species is reported in Table S1.

Here we employ the screening model proposed in Refs. [19, 62]

$$\varepsilon_{eff} = \frac{1 + \sqrt{\varepsilon_{DFPT}}}{2} \ , \tag{S7}$$

with $\varepsilon_{DFPT} = 6.31$.

III. LOOKING FOR A METASTABLE FERROELECTRIC STATE IN THE FE-STRAIN DIAGRAM

The transition into a permanent ferroelectric state implies the presence a metastable state in the free energy surface, corresponding to a vanishing strain condition. In order to verify whether such a local minimum exists, we perform SSCHA calculations at 0K for a 20-atom STO cell with isotropic strains ranging from -0.3% to 0.9%. The order parameter describing the ferroelectric transition is chosen as

$$OP = \left|\sum_i r_{iz}^{crystal}(\varepsilon) - \sum_i r_{iz}^{crystal}(\varepsilon = 0)\right| \ . \tag{S8}$$

Here, $r_{iz}^{crystal}(\varepsilon)$ denotes the crystal coordinate of the i-th atom along the z-direction following a SSCHA relaxation calculation at a strain ε. This parameter is expected to remain approximately constant for a paraelectric system due to inversion symmetry and to change when this symmetry is broken. Fig. S4a represents the order parameter as a function of the strain. We can clearly see that $\varepsilon = 0.6\%$ marks the onset of a ferrolectric transition. This transition corresponds to a change in the free energy profile and the slope of the stress-strain curve (represented in Fig. S4b and Fig. S4c respectively). Remarkably, after the trasition, both the stress-strain curve and the free energy profile continue to change monotonically, making it impossible for the stress to vanish and for the free energy to exhibit a local minimum as a function of the strain. This result implies that there does not exist a metastable ferroelectric state with a periodicity equal to that of a 20-atom STO cell. The strain at which the transition occurs is **largely overestimated** relative to experiment due to the use of the PBE functional for training the MLIP. As mentioned in the main text, this functional tends to underestimate the energy barrier height, thus favoring the paraelectric phase. The predicted transition strain must be corrected using a one-dimensional model of a particle in a double-well potential, given by the equation [63]:

$$\frac{V}{V_0} = -2\left(\frac{x}{x_0}\right)^2 + \left(\frac{x}{x_0}\right)^4 . \tag{S9}$$

The parameters of the model, namely the height V_0 of the energy barrier and the position x_0 of the minima, are tuned based on the RPA calculations from Ref. [18], with values $V_0 = 5$ meV (f.u.) and $x_0 = 0.42\mathring{A}\sqrt{u}$ (f.u.). Fig. S5 shows both the order parameter (solid line) and the pressure (dashed line) obtained by solving the one-dimensional model with SSCHA. The parametrization of the model with RPA calculations (red line) yields a transition strain of 0.1% and a transition pressure of a few hundred kilobars. The accuracy of the model can be evaluated by comparing the predictions obtained from the model parameterized using DFT-PBE calculations (blue line) with the full SSCHA calculation using the MLIP parameterized on PBE calculations in Figs. S4a and S4c. The results show very good agreement, suggesting that a full SSCHA calculation with a MLIP parametrized on RPA calculations would lead a similar result to that obtained with the model (red curve).

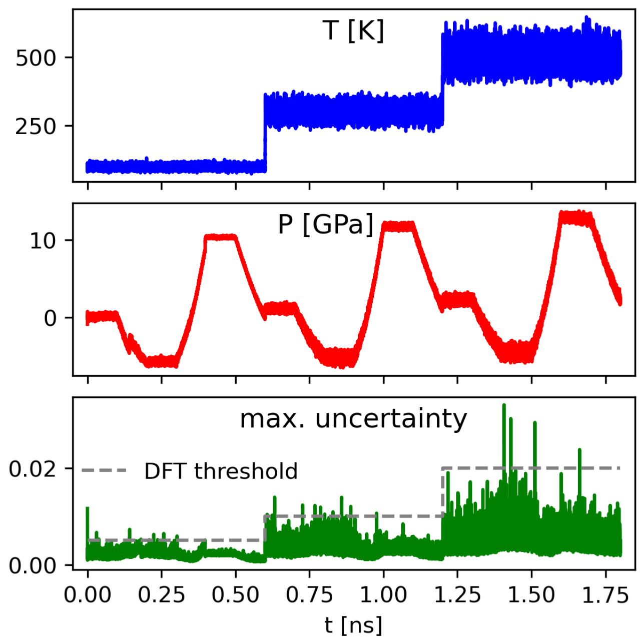

FIG. S1: **Active learning run with FLARE**. At each temperature, the cell is first kept at the DFT-relaxed lattice parameter for 100 ps, then the lattice parameter is linearly increased by 2% over 100 ps, where it is kept constant for another 100 ps. Over the next 100 ps, the lattice parameter is decreased to 2% less than the initial, relaxed value, where it is kept for another 100 ps. Finally, the lattice parameter is returned to the relaxed value over the final 100 ps.

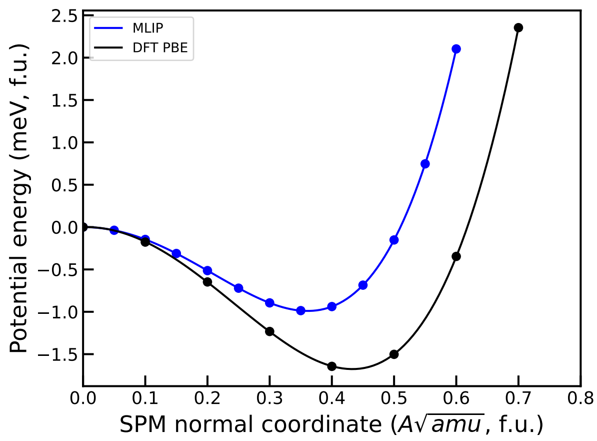

FIG. S2: **PES of the SPM mode**. Potential energy surface of the SPM, calculated both with DFT-PBE (black line) and MLIP (blue line).

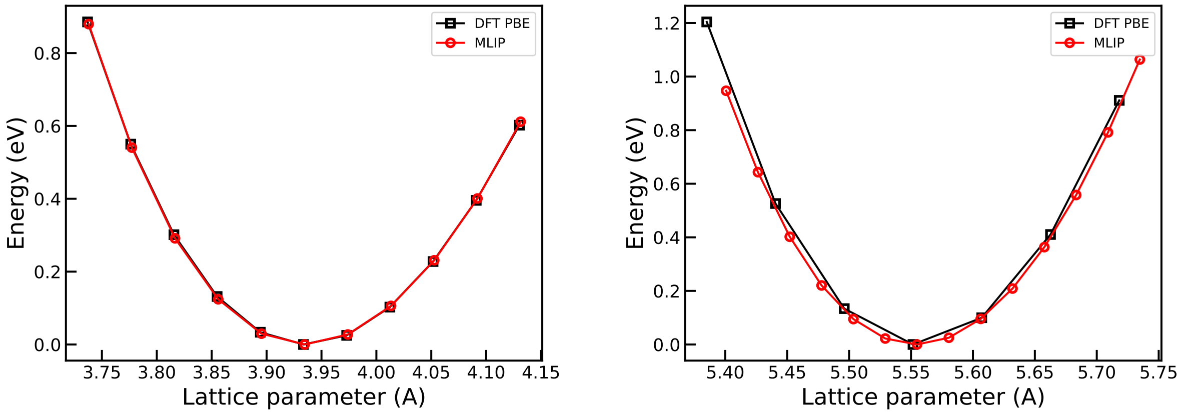

FIG. S3: **Equation of state**. This figure compares the equation of state for the cubic STO cell (Panel a) and the tetragonal 20-atom cell (Panel b), calculated with DFT PBE calculations (black line) with that obtained through MLIP (red line).

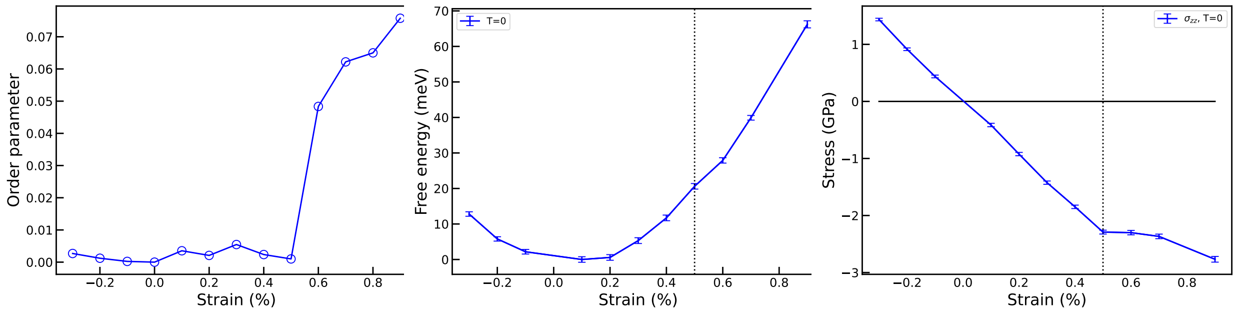

(a) Order parameter (b) Free energy surface (c) Stress

FIG. S4: **SSCHA calculations as a function of strain**. Panel (a) shows the order parameter defined in Eq. S8 as a function of the isotropic strain. Panel (b) shows the zz component of the stress and the free energy (upper and lower part respectively) as a function of the strain. The stress is defined such that a negative value corresponds to tensile stress.

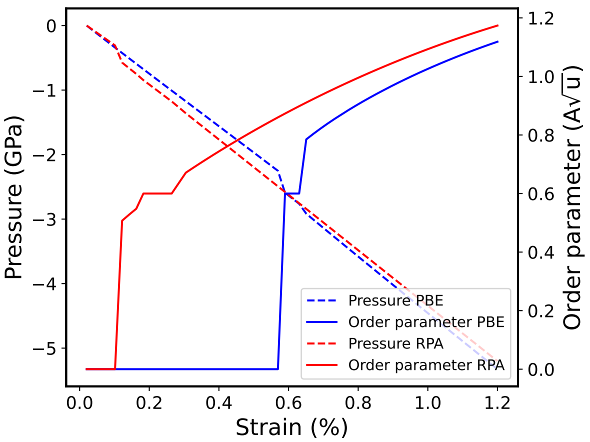

FIG. S5: **Model results**. The solid lines represent the order parameter, indicated by the relaxed position of the particle in the 1D potential, for both the RPA (red) and PBE (blue) parametrizations. The dashed lines depict the pressure acting on the sample, with negative pressure indicating positive stress.

	Z^*_{xx}	Z^*_{yy}	Z^*_{zz}
Ti	7.338	7.338	7.338
Sr	2.549	2.549	2.549
O_1	-2.024	-5.845	-2.024
O_2	-2.024	-2.024	-5.845
O_3	-5.845	-2.024	-2.024

Table S1: **Effective charges**. Born effective charges for the cubic STO unit cell, computed through DFPT.

REFERENCES AND NOTES

1. A. S. Disa, T. F. Nova, A. Cavalleri, Engineering crystal structures with light. *Nat. Phys.* **17**, 1087–1092 (2021).

2. M. F. Becker, A. B. Buckman, R. M. Walser, T. Lépine, P. Georges, A. Brun, Femtosecond laser excitation of the semiconductor-metal phase transition in VO_2. *Appl. Phys. Lett.* **65**, 1507–1509 (1994).

3. A. Cavalleri, C. Tóth, C. W. Siders, J. A. Squier, F. Ráksi, P. Forget, J. C. Kieffer, Femtosecond structural dynamics in VO_2 during an ultrafast solid-solid phase transition. *Phys. Rev. Lett.* **87**, 237401 (2001).

4. X. Li, T. Qiu, J. Zhang, E. Baldini, J. Lu, A. M. Rappe, K. A. Nelson, Terahertz field induced ferroelectricity in quantum paraelectric $SrTiO_3$. *Science* **364**, 1079–1082 (2019).

5. T. F. Nova, A. S. Disa, M. Fechner, A. Cavalleri, Metastable ferroelectricity in optically strained $SrTiO_3$. *Science* **364**, 1075–1079 (2019).

6. A. S. Disa, M. Fechner, T. F. Nova, B. Liu, M. Först, D. Prabhakaran, P. G. Radaelli, A. Cavalleri, Polarizing an antiferromagnet by optical engineering of the crystal field. *Nat. Phys.* **16**, 937–941 (2020).

7. E. J. Sie, C. M. Nyby, C. D. Pemmaraju, S. J. Park, X. Shen, J. Yang, M. C. Hoffmann, B. K. Ofori-Okai, R. Li, A. H. Reid, S. Weathersby, E. Mannebach, N. Finney, D. Rhodes, D. Chenet, A. Antony, L. Balicas, J. Hone, T. P. Devereaux, T. F. Heinz, X. Wang, A. M. Lindenberg, An ultrafast symmetry switch in a weyl semimetal. *Nature* **565**, 61–66 (2019).

8. D. Fausti, R. I. Tobey, N. Dean, S. Kaiser, A. Dienst, M. C. Hoffmann, S. Pyon, T. Takayama, H. Takagi, A. Cavalleri, Light-induced superconductivity in a stripe-ordered cuprate. *Science* **331**, 189–191 (2011).

9. M. Kozina, T. van Driel, M. Chollet, T. Sato, J. M. Glownia, S. Wandel, M. Radovic, U. Staub, M. C. Hoffmann, Ultrafast x-ray diffraction probe of terahertz field-driven soft mode dynamics in $SrTiO_3$. *Struct. Dyn.* **4**, 054301 (2017).

10. M. Kozina, M. Fechner, P. Marsik, T. van Driel, J. M. Glownia, C. Bernhard, M. Radovic, D. Zhu, S. Bonetti, U. Staub, M. C. Hoffmann, Terahertz-driven phonon upconversion in $SrTiO_3$. *Nat. Phys.* **15**, 387–392 (2019).

11. Z. Zhang, F. Y. Gao, Y.-C. Chien, Z.-J. Liu, J. B. Curtis, E. R. Sung, X. Ma, W. Ren, S. Cao, P. Narang, A. von Hoegen, E. Baldini, K. A. Nelson, Terahertz-field-driven magnon upconversion in an antiferromagnet. *Nat. Phys.* **20**, 788–793 (2024).

12. Z. Zhang, F. Y. Gao, J. B. Curtis, Z.-J. Liu, Y.-C. Chien, A. von Hoegen, M. T. Wong, T. Kurihara, T. Suemoto, P. Narang, E. Baldini, K. A. Nelson, Terahertz field-induced nonlinear coupling of two magnon modes in an antiferromagnet. *Nat. Phys.* **20**, 801–806 (2024).

13. M. Basini, M. Pancaldi, B. Wehinger, M. Udina, V. Unikandanunni, T. Tadano, M. C. Hoffmann, A. V. Balatsky, S. Bonetti, Terahertz electric-field-driven dynamical multiferroicity in $SrTiO_3$. *Nature* **628**, 534–539 (2024).

14. D. Kuzmanovski, J. Schmidt, N. A. Spaldin, H. M. Ronnow, G. Aeppli, A. V. Balatsky, Kapitza stabilization of quantum critical order. *Phys. Rev. X* **14**, 021016 (2024).

15. Z. Zhuang, A. Chakraborty, P. Chandra, P. Coleman, P. A. Volkov, Light-driven transitions in quantum paraelectrics. *Phys. Rev. B* **107**, 224307 (2023).

16. G. Shirane, Y. Yamada, Lattice-dynamical study of the 110 °K phase transition in $SrTiO_3$. *Phys. Rev.* **177**, 858–863 (1969).

17. D. Shin, S. Latini, C. Schäfer, S. A. Sato, U. De Giovannini, H. Hübener, A. Rubio, Quantum paraelectric phase of $SrTiO_3$ from first principles. *Phys. Rev. B* **104**, L060103 (2021).

18. C. Verdi, L. Ranalli, C. Franchini, G. Kresse, Quantum paraelectricity and structural phase transitions in strontium titanate beyond density functional theory. *Phys. Rev. Mater.* **7**, L030801 (2023).

19. D. Shin, S. Latini, C. Schäfer, S. A. Sato, E. Baldini, U. De Giovannini, H. Hübener, A. Rubio, Simulating terahertz field-induced ferroelectricity in quantum paraelectric $SrTiO_3$. *Rev. Lett.* **129**, 167401 (2022).

20. L. Ranalli, C. Verdi, L. Monacelli, G. Kresse, M. Calandra, C. Franchini, Temperature-dependent anharmonic phonons in quantum paraelectric $KTaO_3$ by first principles and machine-learned force fields. *Adv. Quantum Technol.* **6**, 2200131 (2023).

21. B. Cheng, P. L. Kramer, Z.-X. Shen, M. C. Hoffmann, Terahertz-driven local dipolar correlation in a quantum paraelectric. *Phys. Rev. Lett.* **130**, 126902 (2023).

22. G. Khalsa, N. A. Benedek, J. Moses, Ultrafast control of material optical properties via the infrared resonant raman effect. *Phys. Rev. X* **11**, 021067 (2021).

23. B. Chen, M. A. Gomez, M. Sehl, J. D. Doll, D. L. Freeman, Theoretical studies of the structure and dynamics of metal/hydrogen systems: Diffusion and path integral Monte Carlo investigations of nickel and palladium clusters. *J. Chem. Phys.* **105**, 9686–9694 (1996).

24. D. Kim, J. D. Doll, J. E. Gubernatis, The quantum dynamics of interfacial hydrogen: Path integral maximum entropy calculation of adsorbate vibrational line shapes for the H/Ni(111) system. *J. Chem. Phys.* **106**, 1641–1645 (1997).

25. G. Krilov, B. J. Berne, Real time quantum correlation functions. II. Maximum entropy numerical analytic continuation of path integral Monte Carlo and centroid molecular dynamics data. *J. Chem. Phys.* **111**, 9147–9156 (1999).

26. G. Baym, N. D. Mermin, Determination of thermodynamic Green's functions. *J. Math. Phys.* **2**, 232–234 (1961).

27. A. Alexandru, G. Başar, P. F. Bedaque, N. C. Warrington, Complex paths around the sign problem. *Rev. Mod. Phys.* **94**, 015006 (2022).

28. L. Mühlbacher, E. Rabani, Real-time path integral approach to nonequilibrium manybody quantum systems. *Phys. Rev. Lett.* **100**, 176403 (2008).

29. D. Marx, M. Parrinello, Ab initio path integral molecular dynamics: Basic ideas. *J. Chem. Phys.* **104**, 4077–4082 (1996).

30. D. M. Ceperley, Path integrals in the theory of condensed helium. *Rev. Mod. Phys.* **67**, 279–355 (1995).

31. D. Chandler, P. G. Wolynes, Exploiting the isomorphism between quantum theory and classical statistical mechanics of polyatomic fluids. *J. Chem. Phys.* **74**, 4078–4095 (1981).

32. I. R. Craig, D. E. Manolopoulos, Quantum statistics and classical mechanics: Real time correlation functions from ring polymer molecular dynamics. *J. Chem. Phys.* **121**, 3368–3373 (2004).

33. T. M. III., D. E. Manolopoulos, Quantum diffusion in liquid water from ring polymer molecular dynamics. *J. Chem. Phys.* **123**, 154504 (2005).

34. F. Libbi, A. Johansson, L. Monacelli, B. Kozinsky, Atomistic simulations of out-of-equilibrium quantum nuclear dynamics. *npj Comput. Mater.* **11**, 102 (2025).

35. L. Monacelli, F. Mauri, Time-dependent self-consistent harmonic approximation: Anharmonic nuclear quantum dynamics and time correlation functions. *Phys. Rev. B* **103**, 104305 (2021).

36. A. Siciliano, L. Monacelli, G. Caldarelli, F. Mauri, Wigner gaussian dynamics: Simulating the anharmonic and quantum ionic motion. *Phys. Rev. B* **107**, 174307 (2023).

37. J. Vandermause, Y. Xie, J. S. Lim, C. J. Owen, B. Kozinsky, Active learning of reactive bayesian force fields applied to heterogeneous catalysis dynamics of h/pt. *Nat. Commun.* **13**, 5183 (2022).

38. Y. Xie, J. Vandermause, L. Sun, A. Cepellotti, B. Kozinsky, Bayesian force fields from active learning for simulation of inter-dimensional transformation of stanene. *npj Comput. Mater.* **7**, 40 (2021).

39. L. Monacelli, R. Bianco, M. Cherubini, M. Calandra, I. Errea, F. Mauri, The stochastic self-consistent harmonic approximation: Calculating vibrational properties of materials with full quantum and anharmonic effects. *J. Phys. Condens. Matter* **33**, 363001 (2021).

40. M. Takesada, M. Itoh, T. Yagi, Perfect softening of the ferroelectric mode in the isotope-exchanged strontium titanate of $SrTi_{18}O_3$ studied by light scattering. *Phys. Rev. Lett.* **96**, 227602 (2006).

41. R. Bianco, I. Errea, L. Paulatto, M. Calandra, F. Mauri, Second-order structural phase transitions, free energy curvature, and temperature-dependent anharmonic phonons in the self-consistent harmonic approximation: Theory and stochastic implementation. *Phys. Rev. B* **96**, 014111 (2017).

42. I. Katayama, H. Aoki, J. Takeda, H. Shimosato, M. Ashida, R. Kinjo, I. Kawayama, M. Tonouchi, M. Nagai, K. Tanaka, Ferroelectric soft mode in a $SrTiO_3$ thin film impulsively driven to the anharmonic regime using intense picosecond terahertz pulses. *Phys. Rev. Lett.* **108**, 097401 (2012).

43. M. Fechner, M. Först, G. Orenstein, V. Krapivin, A. S. Disa, M. Buzzi, A. von Hoegen, G. de la Pena, Q. L. Nguyen, R. Mankowsky, M. Sander, H. Lemke, Y. Deng, M. Trigo, A. Cavalleri, Quenched lattice fluctuations in optically driven $SrTiO_3$. *Nat. Mater.* **23**, 363–368 (2024).

44. L. Monacelli, I. Errea, M. Calandra, F. Mauri, Pressure and stress tensor of complex anharmonic crystals within the stochastic self-consistent harmonic approximation. *Phys. Rev. B* **98**, 024106 (2018).

45. E. Wigner, On the quantum correction for thermodynamic equilibrium. *Phys. Rev.* **40**, 749–759 (1932).

46. K. Imre, E. Özizmir, M. Rosenbaum, P. F. Zweifel, Wigner method in quantum statistical mechanics. *J. Math. Phys.* **8**, 1097–1108 (1967).

47. I. Errea, F. Belli, L. Monacelli, A. Sanna, T. Koretsune, T. Tadano, R. Bianco, M. Calandra, R. Arita, F. Mauri, J. A. Flores-Livas, Quantum crystal structure in the 250-kelvin superconducting lanthanum hydride. *Nature* **578**, 66–69 (2020).

48. R. Bianco, L. Monacelli, M. Calandra, F. Mauri, I. Errea, Weak dimensionality dependence and dominant role of ionic fluctuations in the charge-density-wave transition of $NbSe_2$. *Phys. Rev. Lett.* **125**, 106101 (2020).

49. U. Aseginolaza, R. Bianco, L. Monacelli, L. Paulatto, M. Calandra, F. Mauri, A. Bergara, I. Errea, Phonon collapse and second-order phase transition in thermoelectric SnSe. *Phys. Rev. Lett.* **122**, 075901 (2019).

50. L. Monacelli, N. Marzari, First-principles thermodynamics of $CsSnI_3$. *Chem. Mater.* **35**, 1702–1709 (2023).

51. L. Monacelli, I. Errea, M. Calandra, F. Mauri, Black metal hydrogen above 360 GPa driven by proton quantum fluctuations. *Nat. Phys.* **17**, 63–67 (2021).

52. A. Yamanaka, M. Kataoka, Y. Inaba, K. Inoue, B. Hehlen, E. Courtens, Evidence for competing orderings in strontium titanate from hyper-raman scattering spectroscopy. *Europhys. Lett.* **50**, 688–694 (2000).

53. J. Vandermause, S. B. Torrisi, S. Batzner, Y. Xie, L. Sun, A. M. Kolpak, B. Kozinsky, On-the-fly active learning of interpretable bayesian force fields for atomistic rare events. *npj Comput. Mater.* **6**, 20 (2020).

54. A. Johansson, Y. Xie, C. J. Owen, J. S. Lim, L. Sun, J. Vandermause, B. Kozinsky, Micron-scale heterogeneous catalysis with Bayesian force fields from first principles and active learning. arXiv:2204.12573 [physics.comp-ph] (2022).

55. A. P. Thompson, H. M. Aktulga, R. Berger, D. S. Bolintineanu, W. M. Brown, P. S. Crozier, P. J. in't Veld, A. Kohlmeyer, S. G. Moore, T. D. Nguyen, R. Shan, M. J. Stevens, J. Tranchida, C. Trott, S. J. Plimpton, LAMMPS—A flexible simulation tool for particle-based materials modeling at the atomic, meso, and continuum scales. *Comput. Phys. Commun.* **271**, 108171 (2022).

56. P. Giannozzi, S. Baroni, N. Bonini, M. Calandra, R. Car, C. Cavazzoni, D. Ceresoli, G. L. Chiarotti, M. Cococcioni, I. Dabo, A. D. Corso, S. de Gironcoli, S. Fabris, G. Fratesi, R.

Gebauer, U. Gerstmann, C. Gougoussis, A. Kokalj, M. Lazzeri, L. Martin-Samos, N. Marzari, F. Mauri, R. Mazzarello, S. Paolini, A. Pasquarello, L. Paulatto, C. Sbraccia, S. Scandolo, G. Sclauzero, A. P. Seitsonen, A. Smogunov, P. Umari, R. M. Wentzcovitch, Quantum espresso: A modular and open-source software project for quantum simulations of materials. *J. Phys. Condens. Matter* **21**, 395502 (2009).

57. G. Prandini, A. Marrazzo, I. E. Castelli, N. Mounet, N. Marzari, Precision and efficiency in solid-state pseudopotential calculations. *npj Comput. Mater.* **4**, 72 (2018).

58. R. M. Pick, M. H. Cohen, R. M. Martin, Microscopic theory of force constants in the adiabatic approximation. *Phys. Rev. B* **1**, 910–920 (1970).

59. W. Cochran, R. Cowley, Dielectric constants and lattice vibrations. *J. Phys. Chem. Solid* **23**, 447–450 (1962).

60. S. Baroni, S. de Gironcoli, A. Dal Corso, P. Giannozzi, Phonons and related crystal properties from density-functional perturbation theory. *Rev. Mod. Phys.* **73**, 515–562 (2001).

61. N. Rivano, N. Marzari, T. Sohier, Infrared-active phonons in one-dimensional materials and their spectroscopic signatures. *npj Comput. Mater.* **9**, 194 (2023).

62. K. Yabana, T. Sugiyama, Y. Shinohara, T. Otobe, G. F. Bertsch, Time-dependent density functional theory for strong electromagnetic fields in crystalline solids. *Phys. Rev. B* **85**, 045134 (2012).

63. T. Esswein, N. A. Spaldin, Ferroelectric, quantum paraelectric, or paraelectric? calculating the evolution from $BaTiO_3$ to $SrTiO_3$ to $KTaO_3$ using a single-particle quantum mechanical description of the ions. *Phys. Rev. Res.* **4**, 033020 (2022).
